# Supplementary material for: Negative Impacts of Sleep–Wake Rhythm Disturbances on Attention in Young Adults
Source: Brain Sci. 2022 Nov 30;12(12):1643. doi: 10.3390/brainsci12121643 (PMC9776066; doi:10.3390/brainsci12121643)
Supplement: Supplementary file 1 [file brainsci-12-01643-s001.zip › brainsci-1982632-supplementary.pdf]

Supplementary Materials Table S1. Information of Sleeptime and waketime.

Subject		ID	Day	Sleeptime	Waketime		Subject	Sleeptime	Waketime
1		4	1	00:25	08:15		30	23:10	07:30
2		4	2	00:30	08:15		30	23:00	07:30
3		4	3	00:25	08:00		30	22:45	07:30
4		4	4	23:55	07:45		30	00:00	07:35
5		4	5	00:20	07:30		30	22:50	07:35
6		5	1	00:28	08:20		31	00:10	07:50
7		5	2	00:12	08:10		31	00:05	07:20
8		5	3	23:49	07:02		31	23:50	07:50
9		5	4	23:42	07:20		31	23:30	07:30
10		5	5	23:47	07:21		31	00:10	08:00
11		6	1	01:00	07:50		32	00:15	07:55
12		6	2	00:05	07:45		32	00:10	07:45
13		6	3	00:10	07:50		32	00:20	07:45
14		6	4	00:20	08:10		32	00:21	07:50
15		6	5	00:15	07:50		32	01:00	07:40
16		7	1	00:45	08:15		33	23:55	07:20
17		7	2	00:15	07:15		33	23:50	07:35
18		7	3	00:45	07:20		33	23:50	06:20
19		7	4	00:15	07:30		33	00:00	07:45
20		7	5	00:15	07:50		33	23:45	07:35
21		8	1	01:10	09:15		34	23:55	07:35
22	Experimental Group	8	2	00:50	08:50	Control Group	34	23:45	07:48
23		8	3	00:15	08:10		34	00:05	07:55
24		8	4	00:00	07:25		34	00:00	07:35
25		8	5	00:10	08:40		34	00:20	07:50
26		9	1	00:30	07:55		35	23:20	07:20
27		9	2	00:25	07:30		35	23:25	07:15
28		9	3	00:10	07:15		35	23:55	07:25
29		9	4	00:25	07:30		35	23:45	06:50
30		9	5	00:15	07:30		35	22:50	07:20
31		10	1	02:00	10:15		36	00:05	07:40
32		10	2	01:14	09:15		36	00:10	07:30
33		10	3	00:10	08:00		36	00:20	07:25
34		10	4	00:20	07:55		36	23:35	07:20
35		10	5	00:20	07:45		36	00:05	07:25
36		11	1	00:40	06:15		37	23:30	07:00
37		11	2	00:40	06:15		37	23:10	07:25
38		11	3	00:15	05:15		37	23:55	07:20
39		11	4	00:05	07:50		37	23:50	07:30
40		11	5	01:00	07:50		37	23:00	07:05
41		12	1	01:25	09:35		38	00:08	07:40
42		12	2	00:55	09:15		38	23:52	07:40
43		12	3	00:35	09:05		38	00:02	07:40
44		12	4	00:40	08:50		38	00:00	07:40
45		12	5	01:10	09:00		38	00:12	07:35
46		13	1	02:00	11:10		39	23:15	07:30
47	Experimental Group	13	2	01:50	07:25	Control Group	39	23:55	07:00
48		13	3	01:20	09:50		39	00:05	07:00

49		13	4	01:30	10:00		39	23:30	07:00
50		13	5	01:20	09:05		39	01:55	07:00
51		14	1	00:05	07:45		40	23:00	06:30
52		14	2	23:50	07:45		40	23:00	06:35
53		14	3	00:55	08:00		40	23:30	06:35
54		14	4	00:20	08:30		40	00:00	06:35
55		14	5	00:35	07:45		40	00:00	06:35
56		15	1	01:10	09:08		41	23:38	07:28
57		15	2	00:45	09:00		41	23:55	07:28
58		15	3	00:35	07:30		41	23:58	07:28
59		15	4	00:30	09:06		41	23:53	07:28
60		15	5	00:30	07:20		41	23:53	07:28
61		16	1	01:05	09:30		42	23:33	07:16
62		16	2	00:40	09:05		42	01:30	09:05
63		16	3	00:35	07:30		42	23:56	08:11
64		16	4	00:40	08:30		42	23:50	07:07
65		16	5	00:40	08:30		42	23:15	06:18
66		17	1	00:15	07:30		43	23:57	06:26
67		17	2	00:15	08:45		43	23:46	07:33
68		17	3	00:30	09:00		43	23:56	07:15
69		17	4	00:20	08:45		43	23:59	07:33
70		17	5	00:10	08:45		43	23:59	08:05
71		18	1	01:24	09:30		44	23:50	07:05
72		18	2	01:10	07:30		44	23:19	07:00
73		18	3	00:35	09:00		44	23:17	07:50
74		18	4	00:20	06:30		44	00:10	07:49
75		18	5	00:15	08:45		44	00:15	07:45
76		19	1	00:20	08:30		45	00:00	06:50
77		19	2	00:10	08:40		45	00:15	07:55
78		19	3	00:15	08:30		45	00:10	07:20
79		19	4	00:20	08:30		45	00:05	07:50
80		19	5	00:10	08:20		45	23:55	06:35
81		20	1	01:35	10:00		46	23:55	07:50
82		20	2	02:30	07:56		46	00:15	08:00
83		20	3	01:00	08:20		46	00:20	08:00
84		20	4	00:30	08:25		46	00:00	08:00
85		20	5	01:00	07:50		46	00:20	08:00
86		21	1	01:00	10:00		47	00:20	08:20
87		21	2	02:20	08:03		47	00:40	08:30
88		21	3	03:00	08:00		47	00:22	08:10
89		21	4	02:00	06:00		47	00:21	08:10
90		21	5	00:39	06:50		47	00:00	08:00
91		22	1	02:00	09:30		48	23:50	07:19
92		22	2	01:00	09:00		48	23:45	07:10
93		22	3	00:40	09:00		48	23:40	07:00
94		22	4	00:30	08:45		48	23:40	07:10
95		22	5	01:00	07:50		48	23:55	06:50
96		23	1	01:10	10:16		49	23:55	07:20
97		23	2	01:15	09:29		49	23:50	07:35
98		23	3	01:35	09:25		49	23:50	07:20
99	**Experimental Group**	23	4	01:25	09:15	**Control Group**	49	23:55	07:45

100		23	5	01:05	07:55		49	23:45	07:35
101		24	1	02:32	07:21				
102		24	2	00:36	09:00				
103		24	3	00:34	08:45				
104		24	4	00:23	08:38				
105		24	5	00:37	09:00				
